# Increased Vascular Endothelial Growth Factor Receptor 2 Levels Are Associated With a Higher Occurrence of Coronary Artery Disease in Patients With Obstructive Sleep Apnea

**DOI:** 10.31083/RCM46087

**Published:** 2026-04-14

**Authors:** Jun Zhu, Danyu Wu, Shengyu Chen, Yiwen Pan, Xiaolu Jiao, Guosheng Fu

**Affiliations:** ^1^Department of Cardiology, Sir Run Run Shaw Hospital, School of Medicine, Zhejiang University, 310016 Hangzhou, Zhejiang, China; ^2^Key Laboratory of Cardiovascular Intervention and Regenerative Medicine of Zhejiang Province, 310016 Hangzhou, Zhejiang, China

**Keywords:** coronary artery disease, obstructive sleep apnea, vascular endothelial growth factor receptor 2, Gensini score, SYNTAX score

## Abstract

**Background:**

Obstructive sleep apnea (OSA) has become a vital risk factor for coronary artery disease (CAD). Vascular endothelial growth factor receptor 2 (VEGFR2) participates in the mediation of atherosclerosis, the main underlying pathophysiological basis of CAD, by promoting angiogenesis and inflammation. Chronic intermittent hypoxia, a characteristic of OSA, can induce VEGFR2 expression. Therefore, this study aimed to examine the association between circulating VEGFR2 levels and CAD in OSA patients, an association that has not been well explored in previous research.

**Methods:**

This cross-sectional study involved 453 Chinese adults: 345 with OSA and CAD and 108 with OSA alone. The Gensini and SYNTAX scores were used to evaluate the severity of CAD. An enzyme-linked immunosorbent assay (ELISA) was used to measure circulating VEGFR2 levels. Multivariate logistic regression analyses were used to explore the association between the circulating VEGFR2 levels and CAD and also to determine the independent associations. Multivariate linear regression analysis was used to determine the relationship between VEGFR2 levels and the severity of CAD.

**Results:**

Patients with OSA and CAD demonstrated remarkably higher circulating VEGFR2 levels compared with those patients with OSA alone (median interquartile range (IQR): CAD 10.9 (8.26–14.6) vs. non-CAD 8.25 (5.87–10.3) ng/mL; *p < 0.05). After confounding factors were adjusted, the circulating VEGFR2 level exhibited an independent association with CAD (odds ratio (OR) = 1.17, 95% confidence interval (CI) 1.09–1.27; *p* < 0.001). Furthermore, we confirmed a positive association between VEGFR2 levels and CAD severity in Chinese patients with OSA.

**Conclusions:**

In Chinese patients with OSA, those with CAD exhibited higher circulating VEGFR2 levels than those without CAD. Increased VEGFR2 levels were independently associated with the presence and severity of CAD, suggesting a potential role of VEGFR2 as a biomarker for vascular endothelial dysfunction.

## 1. Introduction

Obstructive sleep apnea (OSA) has a high prevalence rate and impacts about one billion individuals around the world; hence, OSA represents an increasingly serious global health concern [1]. The disorder features the upper airway being recurrently collapsed, accompanied by intermittent hypoxia, hypercapnia, and sleep fragmentation [2], accordingly, inducing sympathetic activation, oxidative stress, metabolic deregulation, and a higher risk of cardiovascular disease (CVD) [3]. According to clinical expert consensus documents, OSA could represent a risk factor or an emerging risk factor for hypertension, diabetes, stroke, atrial fibrillation (AF), heart failure (HF), and coronary artery disease (CAD) [4, 5, 6]. The incidence of CAD in patients with OSA is approximately 20–30% [7]. According to observational studies, untreated OSA triggers a higher risk of revascularization, cardiovascular death, etc. [8, 9]. Continuous positive airway pressure (CPAP) has been validated as being able to effectively relieve relevant symptoms and improve the quality of life of patients; however, CPAP does not reduce acute cardiovascular events in patients with CVD [6, 10], possibly because of patients poorly adhering to CPAP therapy (average usage time: 2.8–3.5 h/night) [11]. Thus, identifying a new target to protect cardiovascular comorbidities in patients with OSA is necessary.

Vascular endothelial growth factor receptor 2 (VEGFR2), also named fetal liver kinase-1 in mice and kinase insert domain-containing receptor in humans, serves as a high-affinity tyrosine kinase receptor for vascular endothelial growth factor (VEGF) [12]. Atherosclerosis is the main underlying pathophysiological basis for CAD [13]. Meanwhile, VEGFR2 is reportedly involved in the growth of atherosclerotic lesions, the presence of unstable plaques, and the formation of negative clinical outcomes by promoting inflammation [14, 15, 16]. Chronic intermittent hypoxia (CIH), a characteristic of OSA, can also induce VEGFR2 expression [17, 18]. Nevertheless, current studies have not fully elucidated the relationship between circulating VEGFR2 levels and CAD in patients with OSA.

Given that VEGFR2 may serve as a new biomarker for patients with OSA at high risk of CAD, this study aimed to examine the relationship between circulating VEGFR2 levels and CAD in these patients.

## 2. Materials and Methods

### 2.1 Subjects

This study was a cross-sectional study, with the study flowchart illustrated in **Supplementary Fig. 1**. A total of 1104 patients aged ≥18 years were admitted to the Department of Cardiology of Sir Run Run Shaw Hospital for coronary angiography from April 2023 to September 2024. Patients with respiratory disease and a requirement for medication including chronic obstructive pulmonary disease (COPD), asthma, interstitial lung disease, and bronchiectasis, as well as those with acute infectious disease, congestive heart failure (CHF), cancer, abnormal renal function, hepatic dysfunction, current pregnancy, and narcolepsy, idiopathic hypersomnolence, chronic insomnia, or any other sleep disorder diagnosed according to the medical record were excluded. Patients who were administered hypnotics, anxiolytics, sedating antidepressants, anticonvulsants, sedating antihistamines, stimulants, or other medications that may impair the nervous system function were also excluded; 543 individuals remained. Each remaining subject was also arranged to receive an overnight sleep study using a Food and Drug Administration (FDA)-approved level II portable diagnostic device (SOMNOscreen; SOMNOmedics GmbH, Randersacker, Germany). OSA referred to a case with an apnea–hypopnea index (AHI) of ≥5 events per hour (American Academy of Sleep Medicine guidelines) [19, 20, 21]. We excluded patients with non-OSA (n = 90), leaving 453 individuals in our study. In this study, CAD was defined as stenosis ≥50% in the left main coronary artery and >70% in a major epicardial vessel, as in a previous study [7]. The patients were divided into two groups based on the results of the coronary angiography. All individuals provided written informed consent before enrollment. The Medicine Ethics Committee at the Sir Run Run Shaw Hospital approved the protocol (ethical approval number: 2020-591-03).

### 2.2 Coronary Severity Evaluation

Evaluation of the CAD severity relied on the Gensini and SYNTAX scores. The Gensini score grades were assessed as previously described [22, 23]. The SYNTAX score was calculated using an online calculator (https://syntaxscore.org/). Two experienced observers, blinded to baseline characteristics of the participants, independently assessed the coronary angiograms and determined CAD diagnoses and scores. Although inter-observer agreement was not formally quantified, any discrepancies were resolved by consensus.

### 2.3 Anthropometric Measurements and Blood Collection

Anthropometric data were collected, including age, gender, body mass index (BMI) (kg/m^2^), blood pressure, medical history, current medications, and smoking and drinking habits. This study defined overweight as a BMI ≥24 kg/m^2^, smokers were subjects who had been smoking or had stopped smoking for <1 year before enrollment, and drinkers were those who consumed alcohol ≥3 days a week. Dyslipidemia, hypertension, and diabetes were defined according to the medical histories of the subjects.

### 2.4 Blood Sample Preparation and Laboratory Studies

Morning blood samples collected from patients after an overnight period underwent 10 min of centrifugation (3000 rpm, 4 °C). Plasma specimens were subsequently maintained at –80 °C before analysis. Plasma soluble VEGFR2 (sVEGFR2) levels were measured using a commercially available enzyme-linked immunosorbent assay (ELISA) kit (R&D Systems, Inc., Minneapolis, MN, USA), which specifically detects the soluble form of VEGFR2. Intra- and inter-assay coefficients of variation (COVs) were confirmed to be <5% and 7%, respectively. According to the manufacturer’s datasheet, the ELISA kit exhibits negligible cross-reactivity with VEGFR1, VEGFR3, or other related VEGF receptor isoforms, and R&D Systems has previously validated the antibody specificity for human plasma samples. Serum biochemical parameters of triglycerides (TGs), high-density lipoprotein cholesterol (HDL-C) levels, low-density lipoprotein cholesterol (LDL-C), TC, fasting blood glucose (FBG), and others were tested in the Biochemical Laboratory of Sir Run Run Shaw Hospital.

### 2.5 Statistical Analysis

All statistical analyses were performed using SPSS version 20.0 (IBM Corp., Armonk, NY, USA). A two-sided *p*-value <0.05 denotes statistical significance. Continuous variables for normally and asymmetrically distributed data are reported as the mean ± standard deviation (SD) and median (interquartile range), respectively. Numerals (percentages) were used to express categorical variables. The independent Student’s *t*-test or Wilcoxon rank sum test was adopted for the difference analysis in continuous variables, while the chi-squared test and Fisher’s exact test served for categorical variables. The association between VEGFR2 and CAD was evaluated using logistic regression analysis. VEGFR2 level-related receiver operating characteristic (ROC) was used to predict CAD in patients with OSA. Multivariable linear regression analysis was performed to evaluate the association of circulating VEGFR2 levels with the severity of CAD. A natural logarithm (Ln) transformation was used for variables with asymmetric distributions.

Missing data were handled using multiple imputation with fully conditional specification (FCS) implemented in SPSS. The imputation model included all variables used in the multivariable analyses. Five imputed datasets (n = 5) were generated with 20 iterations each, and the pooled estimates were calculated using Rubin’s rules.

## 3. Results

### 3.1 Clinical Characteristics of Participants

**Fig. 1.**
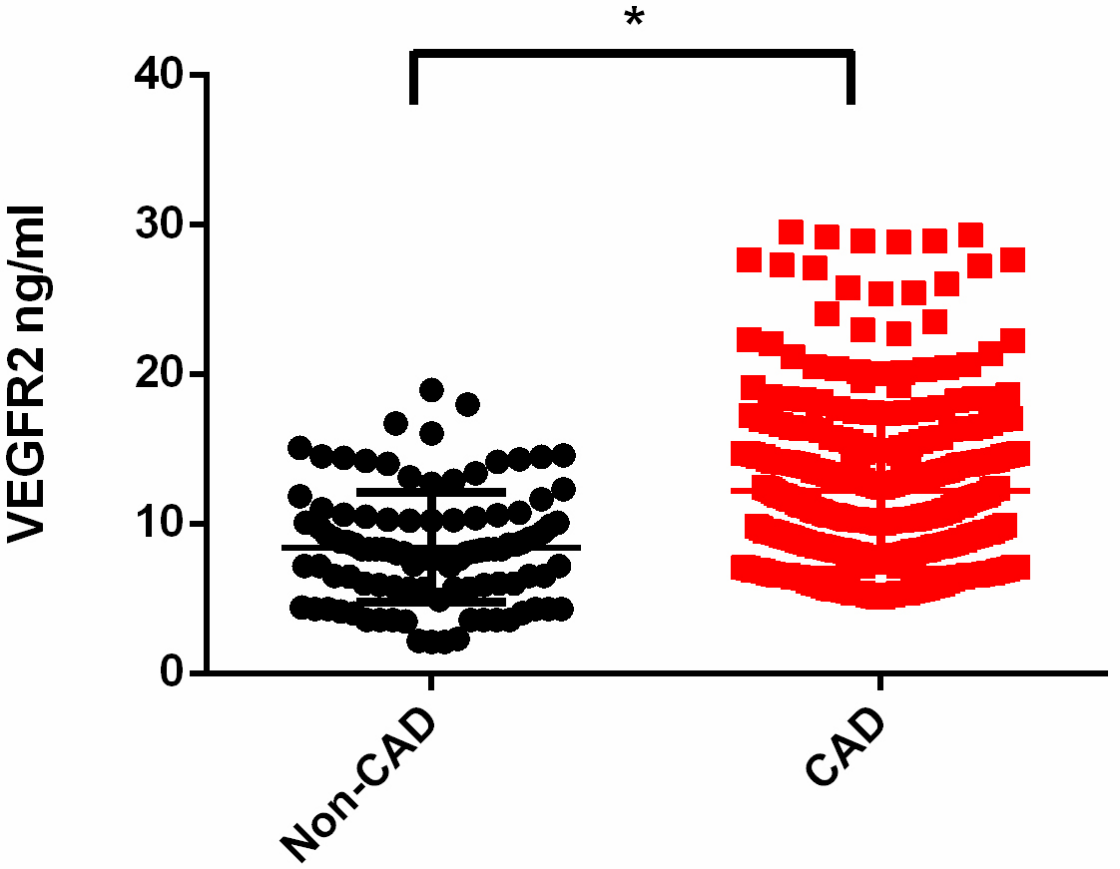
**Increased levels of VEGFR2 in patients with OSA and CAD**. Levels of circulating VEGFR2 in patients with CAD and those without CAD in subjects with OSA (median (IQR): CAD 10.9 (8.26–14.6) vs. non-CAD 8.25 (5.87–10.3) ng/mL; *p < 0.05). Abbreviations: VEGFR2, vascular endothelial growth factor receptor 2; CAD, coronary artery disease; OSA, obstructive sleep apnea; IQR, interquartile range.

A total of 453 individuals with OSA were included in this study: 345 with OSA and CAD and 108 with OSA alone; the clinical features are detailed in Table 1. The two groups did not differ greatly in BMI (*p* = 0.595), TGs (*p* = 0.518), or AHI (*p* = 0.056). CAD patients were likely to be older (*p* < 0.001), and demonstrated remarkably higher FBG (*p* = 0.017), systolic blood pressure (SBP) (*p* < 0.001), diastolic blood pressure (DBP) (*p* < 0.001), TC (*p* < 0.001), and LDL-C levels (*p* < 0.001) versus those without CAD. The HDL-C (*p* < 0.001), average SpO_2_ (*p* = 0.007), and lowest SpO_2_ (*p* = 0.004) levels were lower in CAD patients versus the control group. A larger proportion of CAD individuals were male, smokers, with diabetes and dyslipidemia, versus the non-CAD group. Remarkably, the patients with CAD exhibited a considerably increased level of circulating VEGFR2 compared to the non-CAD group (median interquartile range (IQR): CAD 10.9 (8.26–14.6) vs. non-CAD 8.25 (5.87–10.3) ng/mL; *p < 0.05; Fig. 1). As VEGFR2 levels were not normally distributed, logarithmic transformation was applied for subsequent analyses (**Supplementary Fig. 2**). Furthermore, when stratified by OSA severity, defined as mild (AHI 5–14.9/h), moderate (AHI 15–29.9/h), and severe (AHI ≥30/h), circulating VEGFR2 levels were comparable among mild, moderate, and severe groups (Kruskal–Wallis, *p* = 0.59), whereas CAD prevalence increased with severity (χ^2^ test, *p* = 0.01).

**Table 1.** **Anthropometric and biochemical characteristics of the individuals included in the study**.

	Non-CAD	CAD	*p*-value
	(n = 108)	(n = 345)	
Age (years)	53.56 ± 11.37	56.76 ± 10.00	0.005*
Male (n, %)	76 (70.37%)	304 (88.12%)	<0.001**
BMI (kg/m^2^)	26.56 ± 4.03	26.78 ± 4.52	0.595
Overweight (n, %)	84 (77.78%)	275 (79.71%)	0.666
Smoker (n, %)	38 (35.19%)	176 (51.01%)	0.004*
Drinker (n, %)	37 (34.26%)	122 (35.36%)	0.877
Hypertension (n, %)	73 (67.59%)	213 (61.74%)	0.271
Diabetes (n, %)	11 (10.19%)	82 (23.77%)	0.002*
Dyslipidemia (n, %)	60 (55.56%)	282 (81.74%)	<0.001**
FBG (mmol/L)	6.25 ± 2.09	6.91 ± 2.53	0.017*
SBP (mmHg)	125.37 ± 16.49	135.25 ± 19.79	<0.001**
DBP (mmHg)	75.79 ± 11.26	85.30 ± 15.63	<0.001**
TGs (mmol/L)	1.36 (1.01–1.97)	1.50 (1.06–2.03)	0.518
TC (mmol/L)	4.16 ± 0.96	4.70 ± 1.12	<0.001**
HDL-C (mmol/L)	1.25 ± 0.32	1.04 ± 0.23	<0.001**
LDL-C (mmol/L)	2.48 ± 0.84	2.87 ± 0.91	<0.001**
ALT (U/L)	22.00 (15.00–40.00)	26.00 (19.00–41.00)	0.076
AST (U/L)	22.00 (17.00–29.00)	24.00 (19.00–36.00)	0.003*
Creatinine (µmol/L)	52.58 ± 31.55	70.29 ± 16.12	<0.001**
Uric acid (µmol/L)	366.17 ± 117.94	367.99 ± 88.75	0.692
Gensini Score	NA	32 (12.00–55.00)	
SYNTAX Scores	NA	12.00 (7.00–20.00)	
AHI (times/h)	15.30 (10.33–25.68)	19.10 (10.30–32.90)	0.056
Lowest SpO_2_ (%)	86.74 ± 10.68	84.02 ± 7.63	0.004*
Average SpO_2_ (%)	94.84 ± 3.55	93.34 ± 5.43	0.007*

Mean ± standard deviation, median (interquartile range), or n (%) is used to express the results. Independent Student *t*-test, Fisher’s exact test, χ^2^ test, or Wilcoxon test is used to analyze the differences between groups. Abbreviations: BMI, body mass index; CAD, coronary artery disease; FBG, fasting blood glucose; SBP, systolic blood pressure; DBP, diastolic blood pressure; TGs, triglycerides; TC, total cholesterol; HDL-C, high-density lipoprotein cholesterol; LDL-C, low-density lipoprotein cholesterol; AHI, apnea–hypopnea index; NA, not applicable; ALT, alanine aminotransferase; AST, aspartate aminotransferase. *p < 0.05, **p < 0.001.

### 3.2 Association Between Circulating VEGFR2 Level and CAD

Different logistic regression models were constructed. Univariate analysis showed that higher circulating VEGFR2 levels were associated with a higher odds ratio (OR) for CAD (OR = 1.24, 95% CI 1.16–1.33; *p* < 0.001; Table 2). Additionally, multivariate analysis revealed that after conventional CAD risk factors (age, gender, BMI, smoking habitats, drinking habitats, SBP, DBP, LDL-C, HDL-C, TC, TGs, FBG, creatinine, medication use including statins, antiplatelets, beta-blockers, and angiotensin-converting enzyme (ACE) inhibitors/angiotensin II receptor blockers (ARBs), and oxygen saturation indices including lowest SpO_2_, average SpO_2_, time below 90% oxygen saturation (T90), and oxygen desaturation index (ODI)) were adjusted, elevated levels of VEGFR2 showed an independent relevance to the presence of CAD (OR = 1.17, 95% CI 1.09–1.27; *p* < 0.001).

**Table 2.** **Multivariate logistic regression analyses of circulating VEGFR2 levels and presence of CAD in patients with OSA**.

	Unadjusted		Model 1		Model 2	
	OR (95% CI)	*p*-value	OR (95% CI)	*p*-value	OR (95% CI)	*p*-value
Ln–VEGFR2	1.24 (1.16–1.33)	<0.001**	1.24 (1.16–1.33)	<0.001**	1.17 (1.09–1.27)	<0.001**
T1 (n = 151)	Reference		Reference		Reference	
T2 (n = 151)	2.93 (1.76–4.97)	<0.001**	3.01 (1.76–5.23)	<0.001**	2.00 (1.02–3.99)	0.045*
T3 (n = 151)	5.49 (3.07–10.26)	<0.001**	5.51 (3.01–10.53)	<0.001**	3.22 (1.55–6.92)	0.002*

Model 1: adjusted for age, gender, BMI, smoking habits, and drinking habits. Model 2: adjusted for model 1 + SBP, DBP, FBG, TGs, TC, HDL-C, and LDL-C, creatinine, medication use (statins, antiplatelets, beta-blockers, and ACE inhibitors/ARBs), and oxygen saturation indices (lowest SpO_2_, average SpO_2_, T90, and ODI). Tertile values of VEGFR2 are expressed as T1 (≤8.33 ng/mL), T2 (8.35–12.6 ng/mL), and T3 (>12.6 ng/mL). Abbreviations: VEGFR2, vascular endothelial growth factor receptor 2; CAD, coronary artery disease; OSA, obstructive sleep apnea; OR, odds ratio; CI, confidence interval; BMI, body mass index; SBP, systolic blood pressure; DBP, diastolic blood pressure; FBG, fasting blood glucose; TGs, triglycerides; TC, total cholesterol; HDL-C, high-density lipoprotein cholesterol; LDL-C, low-density lipoprotein cholesterol; T90, time below 90% oxygen saturation; ODI, oxygen desaturation index; ACE, angiotensin-converting enzyme; ARBs, angiotensin II receptor blockers. *p < 0.05, **p < 0.001.

Furthermore, the relationship between VEGFR2 levels and CAD severity was assessed using multivariate linear regression. Because the Gensini and SYNTAX scores were not normally distributed, logarithmic transformation was applied before the regression analyses (**Supplementary Fig. 3**). After the abovementioned CAD risk factors were adjusted, the circulating VEGFR2 level exhibited a positive association with the Gensini score (β = 1.09, 95% CI 0.77–1.42; *p* < 0.001) and the SYNTAX score (β = 0.77, 95% CI 0.53–1.01; *p* < 0.001), which explains the severity of CAD (Table 3).

**Table 3.** **Multivariate linear regression analyses of circulating VEGFR2 levels and severity of CAD**.

	Unadjusted		Model 1		Model 2	
	β (95% CI)	*p*-value	β (95% CI)	*p*-value	β (95% CI)	*p*-value
Ln–Gensini	1.52 (1.20–1.84)	<0.001**	1.47 (1.15–1.79)	<0.001**	1.09 (0.77–1.42)	<0.001**
Ln–SYNTAX	1.08 (0.84–1.32)	<0.001**	1.04 (0.81–1.28)	<0.001**	0.77 (0.53–1.01)	<0.001**

Dependent variable: Ln-transformed–VEGFR2. Model 1: adjusted for age, gender, BMI, smoker, and drinker. Model 2: adjusted for model 1 + SBP, DBP, FBG, TGs, TC, HDL-C, LDL-C, creatinine, medication use (statins, antiplatelets, beta-blockers, and ACEI/ARBs), and oxygen saturation indices (lowest SpO_2_, average SpO_2_, T90, and ODI). Abbreviations: VEGFR2, vascular endothelial growth factor receptor 2; CAD, coronary artery disease; BMI, body mass index; SBP, systolic blood pressure; DBP, diastolic blood pressure; FBG, fasting plasma glucose; TG, triglycerides; TC, total cholesterol; HDL-C, high-density lipoprotein cholesterol; LDL-C, low-density lipoprotein cholesterol; T90, time below 90% oxygen saturation; ODI, oxygen desaturation index. **p < 0.001.

## 4. Discussion

Collectively, patients with CAD and OSA had considerably higher circulating VEGFR2 levels than those with OSA alone. Furthermore, circulating VEGFR2 levels in OSA patients were independently associated with CAD risk and positively correlated with CAD severity.

OSA features the upper airway being frequently collapsed during sleep, accompanied by lower oxygen saturation, hypercapnia, and sleep interruption. OSA is reportedly a condition closely correlated with CVD [18]. For community-dwelling individuals without overt CVD, OSA indicates early-stage atherosclerosis [24] and the presence of coronary plaque burden [25]. OSA develops alongside hypertension, stroke, CAD, HF, and AF, and is also linked to metabolic problems, including obesity, insulin resistance, type-2 diabetes, and metabolic syndrome [26]. CPAP therapy is considered the most effective method for alleviating OSA. According to randomized controlled trials, CPAP therapy excels in improving the quality of sleep, relieving excessive daytime tiredness [6], lowering SBP [27, 28], enhancing endothelial function [29], and strengthening insulin sensitivity [30]; however, CPAP cannot effectively handle cardiovascular events in OSA patients [4, 10]. Thus, identifying new targets to prevent CVD in OSA patients is urgent.

VEGFR2 is the receptor for VEGFs A, C, and D, and plays an essential role in regulating angiogenesis, vascular development, permeability, and embryonic hematopoiesis. VEGFR2 promotes endothelial cell proliferation, migration, differentiation, and survival. Notably, the assay employed in this study measured soluble VEGFR2 (sVEGFR2) rather than the membrane-bound receptor. The soluble form reflects receptor shedding and endothelial activation, which may differ from the signaling function of the membrane-bound VEGFR2. Therefore, the present findings should be interpreted in the context of circulating sVEGFR2 biology. CIH is a prominent feature of OSA, and VEGFR2 levels have been positively associated with CIH severity [17]. Furthermore, CIH has been shown to induce VEGFR2 expression [31].

Atherosclerosis, a prevalent chronic inflammatory disease involving angiogenesis [32], is the main underlying pathophysiological basis for CAD; VEGFR2 is crucially involved in atherosclerosis development [33]. VEGFR2 polymorphisms (rs1870377, rs2071559, and rs2305948) could be employed to help identify individuals at higher risk of atherosclerotic CVD [34]. Activation of VEGFR2 by C1q/tumor necrosis factor-α-related protein 1 might be involved in vascular hyperpermeability under disturbed flow conditions, which may promote the pathogenesis of early-stage atherosclerosis [35]. Augmented focal adhesion kinase activity aggravates disturbed flow-mediated atherosclerosis by providing beneficial effects toward reducing proinflammation via VEGFR2–Cbl–NF-κB signaling [36]. Therefore, blocking VEGFR2 promotes the maturation of nascent vessels, thereby preventing intraplaque hemorrhage and inflammatory cell influx and increasing plaque stability [15]. This study found that circulating VEGFR2 levels were independently associated with CAD after adjusting for confounding factors. Therefore, VEGFR2 possibly serves as a new biomarker and therapeutic target for atherosclerotic CVD in OSA patients, although this remains to be determined in future research. Interestingly, while CAD prevalence increased significantly with OSA severity, circulating VEGFR2 levels remained comparable across mild, moderate, and severe OSA groups. This suggests that an elevation in VEGFR2 levels is unlikely to be driven solely by OSA severity or hypoxic burden. Instead, the increased VEGFR2 levels observed in this study may primarily reflect the presence of CAD and the associated endothelial dysfunction rather than the degree of OSA. Our analysis suggests VEGFR2 may serve as a potential biomarker reflecting vascular dysfunction and atherosclerotic burden in OSA patients. If validated in larger longitudinal cohorts, circulating VEGFR2 could potentially serve as a biomarker to identify OSA patients at higher cardiovascular risk. Such patients might benefit from more intensive management strategies, including strict adherence to CPAP therapy and optimization of anti-atherosclerotic treatments. Future interventional studies are warranted to determine whether targeting VEGFR2-related pathways could improve cardiovascular outcomes in this population.

According to previous studies, age is independently related to CAD, and more individuals with CAD are male [23]. Here, the number of male subjects in the CAD group was higher than in the non-CAD group; hence, age and gender were adjusted for to avoid confounding. Obesity is a pivotal factor influencing CAD; VEGFR2 might be involved in the development of obesity [37] and fat tissue expansion [38]. Dyslipidemia and hyperglycemia crucially drive the pathogenesis of CAD [39, 40]. In our study, patients with CAD exhibited higher BMI, DBP, FBG, and TC, but lower HDL-C, than non-CAD subjects. Meanwhile, BMI, DBP, TC, HDL-C, and FBG were adjusted to avoid confounding effects.

To avoid bias, we carefully followed the recommendations in our previous study [23]. First, the ELISA was administered by a researcher who was trained and unaware of the clinical data of patients, as per the protocol of the producer. Then, the confounding effects of the risk factors were adjusted in the statistical analysis. Finally, the cross-sectional design, coupled with consecutive participant recruitment, reduced the influence of outcome selection bias [41].

To verify whether the sample size was sufficient for the logistic regression analysis, a post hoc power analysis was performed using PASS software (version 11, NCSS, LLC, Kaysville, UT, USA). Based on the observed effect size of VEGFR2 for the presence of CAD in patients with OSA (odds ratio ≈ 1.17 per unit increase in Ln–VEGFR2, α = 0.05), and the total sample size of 453 participants (151 per tertile group), the calculated actual power (1-β) was greater than 0.80. This indicates that the current sample size provided adequate statistical power to detect the observed association between circulating VEGFR2 levels and CAD in OSA patients.

Despite these measures, our study had some limitations. (1) As this was a cross-sectional study, causal relationships between VEGFR2 levels and CAD could not be established. Therefore, it remains uncertain whether elevated VEGFR2 reflects a causal role in CAD development or is a secondary consequence of CAD and its treatment. Prospective longitudinal studies with follow-up for new-onset CAD events are warranted to determine the predictive value of VEGFR2 in this population. (2) Most enrolled individuals were taking medication (**Supplementary Table 1**). Meanwhile, the effects of drugs on the VEGFR2 level were not observed. In addition, inter-reader agreement for angiographic interpretation was not formally assessed, which may introduce a minor degree of classification bias. (3) The controls were also from our cardiology department, rather than the general population. (4) The cohort consisted entirely of patients undergoing coronary angiography at a single tertiary care center. Although this design is clinically reasonable for accurately determining the presence and severity of CAD, it may introduce referral bias, as these participants represent a population with a higher pretest probability of coronary disease. Consequently, our findings may not be directly generalizable to the broader OSA population in outpatient or community settings. (5) All participants in this study were Chinese. As VEGFR2 expression and circulating levels may vary across ethnicities and genetic backgrounds, caution should be taken when generalizing our findings to other populations. Future studies involving multi-ethnic cohorts and assessment of VEGFR2 genetic polymorphisms are warranted. (6) CAD was defined based on angiographic stenosis criteria without further stratification by clinical subtypes (*e*.*g*., stable angina or acute coronary syndrome) or lesion location (*e*.*g*., LAD vs. non-LAD). Future studies with detailed phenotypic classification are warranted to determine whether VEGFR2 is differentially associated with specific CAD subtypes.

## 5. Conclusions

Overall, CAD patients exhibited considerably higher circulating VEGFR2 levels than those without CAD among Chinese individuals with OSA. Elevated VEGFR2 levels were independently associated with the presence and severity of CAD. These findings suggest that VEGFR2 may serve as a potential biomarker reflecting vascular dysfunction and atherosclerotic burden in OSA patients.

## Data Availability

The datasets generated and analyzed during the current study are not publicly available due to patient privacy and institutional regulations but are available from the corresponding author on reasonable request.

## Abbreviations

AHI, apnea–hypopnea index; BMI, body mass index; CAD, coronary artery disease; CIH, chronic intermittent hypoxia; CVD, cardiovascular disease; DBP, diastolic blood pressure; FBG, fasting blood glucose; HDL-C, high-density lipoprotein cholesterol; LDL-C, low-density lipoprotein cholesterol; OSA, obstructive sleep apnea; SBP, systolic blood pressure; SpO_2_, peripheral capillary oxygen saturation; TC, total cholesterol; TGs, triglycerides; VEGFR2, vascular endothelial growth factor receptor 2.

## Supplementary Material

Supplementary material associated with this article can be found, in the online version, at https://doi.org/10.31083/RCM46087.
