# Supplementary material for: Increased Vascular Endothelial Growth Factor Receptor 2 Levels Are Associated With a Higher Occurrence of Coronary Artery Disease in Patients With Obstructive Sleep Apnea
Source: Rev Cardiovasc Med. 2026 Apr 14;27(4):46087. doi: 10.31083/RCM46087 (PMC13155991; doi:10.31083/RCM46087)

Supplementary Table 1. List of medications for participants included in this study.

| medication | CAD | Non-CAD |
| --- | --- | --- |
| Statins | 295 | 54 |
| Ezetimibe | 189 | 23 |
| Diuretic | 96 | 14 |
| ACEI/ARB | 147 | 32 |
| β-blocker | 240 | 6 |
| Calcium channel blockers | 189 | 24 |
| Aspirin | 268 | 48 |
| Clopidogrel | 239 | 39 |
| Nitrates | 136 | 28 |
| Others | 114 | 19 |

**Supplementary Fig. 1.** Flow chart of inclusion of subjects in this study


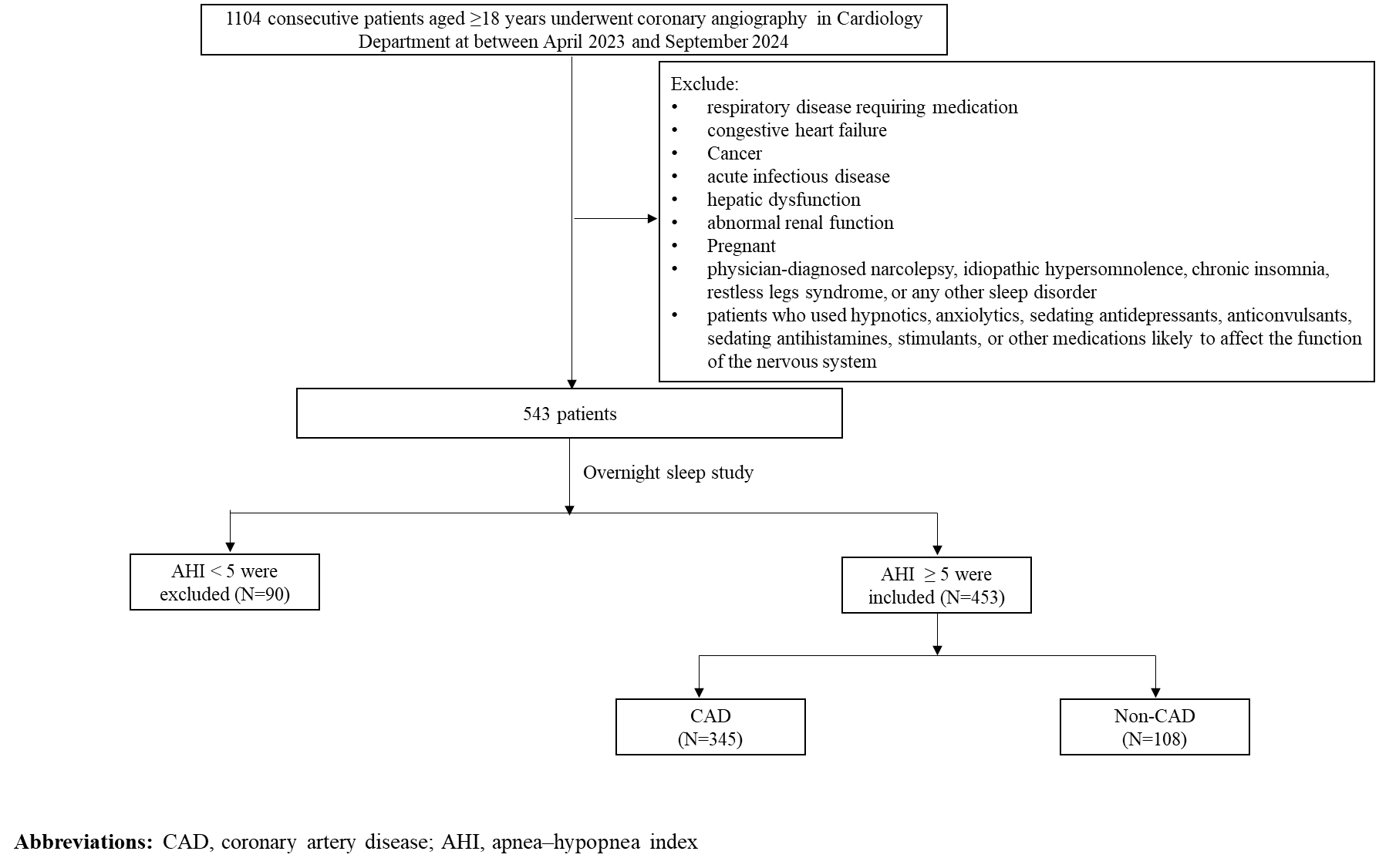


**Supplementary Fig. 2. The distribution plots for VEGFR2 and Ln-transformed VEGFR2**


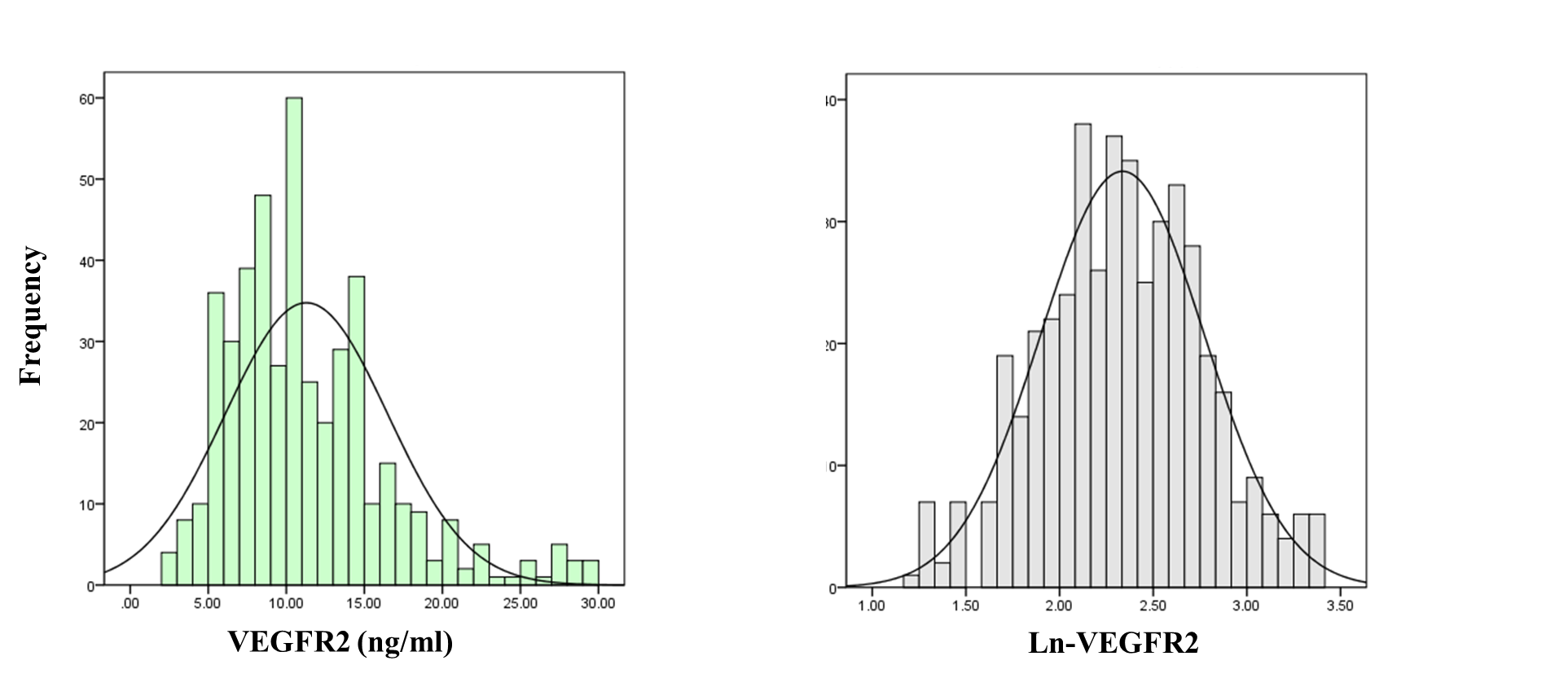


**Supplementary Fig. 3. The distribution plots for Ln-transformed Gensini and SYNTAX scores**
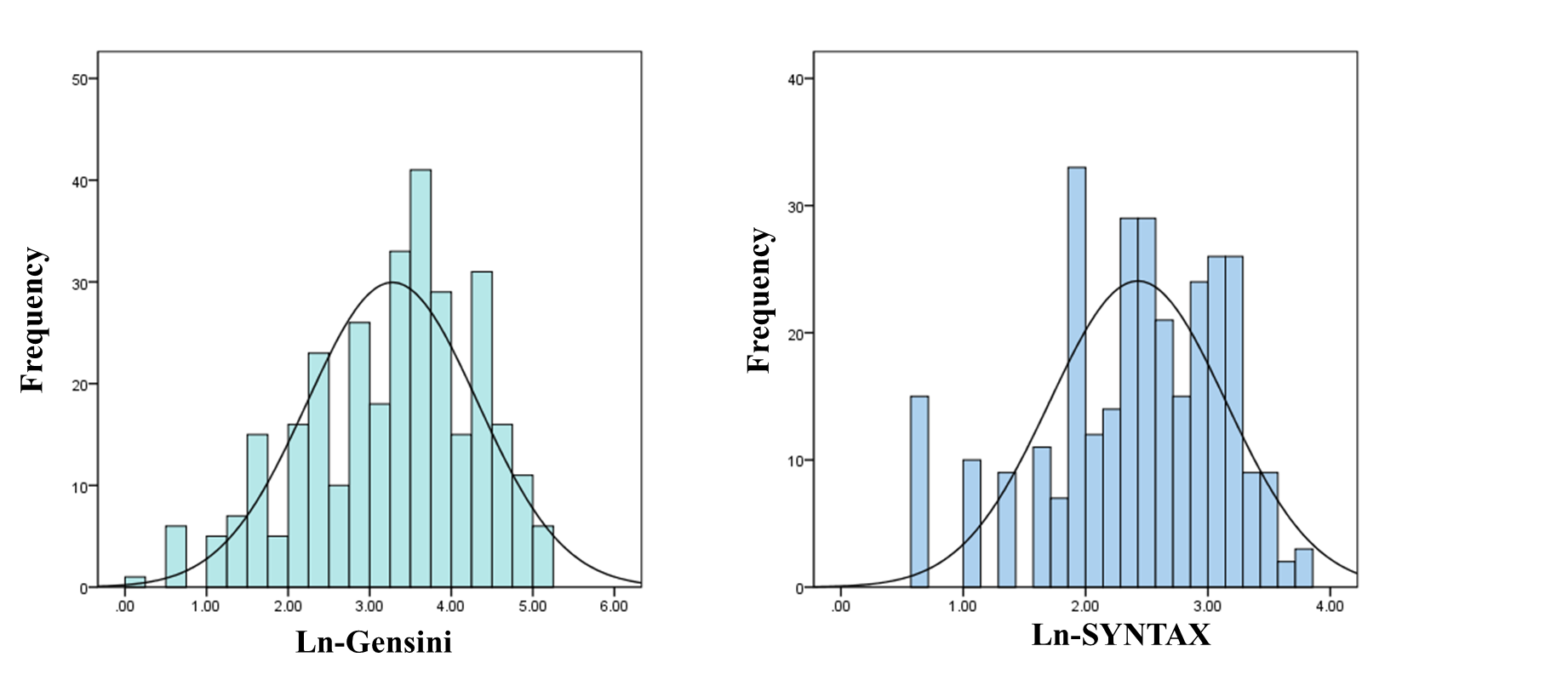

Supplement: Supplementary file 1 [file 2153-8174-27-4-46087-s1.zip › Supplementary Material.docx]
